# The large-sized darter *Anhinga pannonica* (Aves, Anhingidae) from the late Miocene hominid Hammerschmiede locality in Southern Germany

**DOI:** 10.1371/journal.pone.0232179

**Published:** 2020-05-06

**Authors:** Gerald Mayr, Thomas Lechner, Madelaine Böhme

**Affiliations:** 1 Senckenberg Research Institute and Natural History Museum Frankfurt, Ornithological Section, Frankfurt am Main, Germany; 2 Senckenberg Center for Human Evolution and Paleoecology (HEP), Eberhard-Karls University Tübingen, Institute for Geoscience, Tübingen, Germany; Chinese Academy of Sciences, CHINA

## Abstract

We report fossils of the darter *Anhinga pannonica* Lambrecht, 1916 from two late Miocene (Tortonian, 11.62 and 11.44 Ma) avifaunas in Southern Germany. The material from the hominid locality Hammerschmiede near Pforzen represents the most comprehensive record of this species and includes most major postcranial elements except for the tarsometatarsus. We furthermore show that the putative cormorant *Phalacrocorax brunhuberi* (von Ammon, 1918) from the middle Miocene of Regensburg-Dechbetten is another, previously misclassified, record of *A*. *pannonica*, and this may also be true for early Miocene fossils described as *P*. *intermedius* Milne-Edwards, 1867. *A*. *pannonica* was distinctly larger than extant darters and reached the size of *A*. *grandis* from the late Miocene of North America. We detail that only fossils from the Miocene of Europe and Africa can be referred to *A*. *pannonica*, whereas putative records from Asia fall within the size range of extant darters. *A*. *pannonica* appears to have been a long-living species (16 to 6 Ma) with an extensive distribution from the equator to the northern mid-latitudes. The extinction of large-sized darters in Europe is likely to have been due to climatic cooling in the late Neogene, but the reasons for their disappearance in Africa and South America remain elusive.

## Introduction

Darters or snakebirds (Anhingidae) are the sister taxon of cormorants (Phalacrocoracidae) and include four extant species of highly aquatic birds, which occur in tropical and subtropical freshwater habitats of the Americas (*Anhinga anhinga*), Africa (*A*. *rufa*), Asia (*A*. *melanogaster*), and the Australian region (*A*. *novaehollandiae*) [1, 2]. Darters are leg-propelled divers, which forage by skewering larger prey items, mainly fishes and aquatic amphibians, with their long and pointed beak.

The fossil record shows that darters were much more diverse in the past and this is particularly true for South America, where species of the taxa *Macranhinga*, *Meganhinga*, and *Giganhinga* reached a very large size and coexisted with smaller darters (*Anhinga minuta* and *A*. *hesterna*) during the Miocene and Pliocene [3–14]. Truly giant darters, some of which were probably flightless [5, 8], were restricted to South America. However a species that was distinctly larger than all extant darters, *Anhinga grandis*, was reported from the late Miocene of Nebraska and Florida [15, 16]; tentative records of *A*. *grandis* were also described from the late Miocene/early Miocene of Brazil [6] and the middle Miocene of Colombia [17]. *A*. *subvolans* from the early Miocene (ca. 18 Ma) of Florida, which is the oldest New World record of the Anhingidae, was somewhat larger than the largest extant Anhingidae but did not reach the size of *A*. *grandis* [18].

The Old World fossil record of darters includes the oldest unambiguously identified fossil species assigned to the clade, *Anhinga walterbolesi* from the late Oligocene or early Miocene (24–26 Ma) of Australia, of which, however, only the tarsometatarsus is known [19](the exact age and phylogenetic placement of *Protoplotus beauforti* from the early Paleogene of Sumatra is controversial [14, 20]). From Australia, several Neogene species of darters were described [21], and darters were also found in the Neogene of Africa and Asia [22–24].

Darters do not occur in Europe today, but the continent yielded one of the first fossil darters to have been described scientifically. This species, *Anhinga pannonica* (Lambrecht, 1916), was established on the basis of a 6^th^ cervical vertebra from the late Miocene (MN 9; ~10 Ma) of Brusturi in Romania (the locality was then part of the Austro-Hungarian Empire and was termed Tataros); a carpometacarpus from the same site was also assigned to *A*. *pannonica* [25, 26]. Various fossils from the Miocene and Pliocene of Africa and Asia have subsequently at least tentatively been referred to *A*. *pannonica*. These include a cervical vertebra and a proximal humerus from the late Miocene Beglia Formation (MN 9; ca. 10–11 Ma) of Tunisia [27], a partial tarsometatarsus and a humerus fragment from the late Miocene of Pakistan [28], fragmentary leg bones from the early Miocene (MN 4; ca 16 Ma) of Thailand [29], a proximal humerus from the middle Miocene (12–13 Ma) Ngorora Formation of Kenya [30], as well as partial humeri from the late Miocene (7 Ma) of Toros-Menalla in Chad [23]. Bones of a large, unidentified darter were also reported from the latest Miocene Sahabi Formation of Libya [31, 32].

The European record of *A*. *pannonica* is much sparser and, in addition to the two bones described by Lambrecht [25], consists of two partial humeri from the late Miocene (MN 9; 9.8 Ma) of Götzendorf in Austria [33] and a proximal humerus from the early middle Miocene (MN 5; 16.0–15.2 Ma) of the Hambach opencast coal mine in Germany [34, 35]. A putative record of a darter from the middle Miocene (MN 6–8; ca. 13.5–11 Ma) of Hungary is only represented by an ungual pedal phalanx [36].

Here we report multiple remains of *Anhinga pannonica*, which significantly add to our knowledge of this species. The fossils stem from the Hammerschmiede clay pit near Pforzen (Allgäu region, Bavaria, Germany). The fossiliferous sediments of this locality were deposited in a subtropical, fluviatile environment during the earliest late Miocene (Tortonian; MN 8). Bird fossils come from the stratigraphic levels Hammerschmiede 4 and 5 (HAM 4 and HAM 5). Both Hammerschmiede levels represent floodplain channels of meandering fluvial systems of different age and dimension [37]. The level HAM 5 (dated to 11.62 Ma) represents a small-sized channel with a width of four to five meters and a channel fill thickness of 0.8–1 meter, corresponding to a rivulet of local origin [38]. The channel dimensions of the stratigraphically younger level HAM 4 (11.44 Ma) indicate a medium-sized river (width ~50 m, thickness 4–5 m). Both channels are asymmetric in cross section with a more deeply incised outer bank and a shallower slip-off slope. Based on the depth of fluvial incision into the bedrock, the mean water depths can be estimated as ≤ 0.8 m for HAM 5 and ≤ 4 m for HAM 4. Based on the grain sizes of the channel fills (HAM 4: clay to fine sandy, HAM 5: clay to very fine sandy), estimated flow velocities were low to very low. Drift wood, sometimes as long as two meters, is commonly observed in the deposits of HAM 4. Even the narrow and shallow rivulet HAM 5 is in agreement with the ecology of extant darters, which are as specialist shallow-water divers with observed dive depths < 0.5 m [39].

The Hammerschmiede locality has long been known for rich vertebrate assemblages [40, 41], and excavations of the past years have significantly augmented the diversity of the known fauna, which is so far represented by more than 120 vertebrate taxa. Most notable among the recent finds are fossils of the arboreal bipedal hominid *Danuvius guggenmosi* [38], but the vertebrate fossil record of the Hammerschmiede locality includes numerous other—from an extant European perspective—unusual vertebrate groups ([37]: Table 1), such as the giant urodele *Andrias scheuchzeri*, the latest records of the archosauromorph taxon Choristodera, and the bear *Kretzoiarctos*, which is a stem group representative of the Giant Panda [42]. Both Hammerschmiede channel fills contain abundant and diverse fish fossils, especially from small to medium-sized species (standard length 10–20 cm), such as true catfish (*Silurus*), cypriniforms (loach, minnows, barbs, and others) and perciforms (perch, goby), indicating that these fluvial systems provided ample food resources for piscivorous darters.

**Table 1:** Dimensions (in millimeters) of selected bones of *Anhinga pannonica* from the Hammerschmiede clay pit in comparison to fossil and extant Anhingidae (of *Giganhinga kikuyensis* and *Meganhinga chilensis* no comparable measurements were published, but these species are much larger than *A*. *pannonica*).

	Humerus, length	Humerus, midshaft width	Tibiotarsus, distal width	Carpometacarpus, length	Femur, length
†*Anhinga pannonica*	157.5	7.8	12.0	~77	~65
*A*. *rufa*	128.7–132.0 [23]	6.2–7.9 [18]	10.4–11.0 [22]	63.0; 69.3–71.0 [22]	55.3–59.2 [22]
*A*. *melanogaster*	133.1–140.9 [23]	6.4 [18]	N/A	N/A	56.5
*A*. *novaehollandiae*	137.0 [63]	7.0 [18]	12.2 [6]	72.6 [63]	58.0 [6]
*A*. *anhinga*	113.2–137.6 [16]	5.7–7.1 [18]	9.6–10.9 [16]	59.8–68.4 [16]	55.0–59.2 [6, 22]
†*A*. *minuta*	99.0 [6]	5.4 [6]	8.5 [6]	—	—
†*A*. *subvolans*	—	7.6 [18]	—	—	—
†*A*. *grandis*	~150 (est.) [16]	7.8–9.6 [16]	11.4 [16]	74.8 [16]	—
†*Macranhinga paranensis*	176.0–180 [4, 63]	10.2 [4]	20.0 [4]	81.2–84.4 [4]	87.0 [4]
†*M*. *ranzii*	—	—	—	—	95.2–~100 [6]
†*M*. (“*Anhinga*”) cf. *fraileyi*	~135.0 (est.) [6]	8.0 [6]	—	—	—

Extinct species are indicated by a dagger; unlabeled measurements are based on skeletons in the collection of Senckenberg Research Institute Frankfurt; references for values from the literature are given in brackets. N/A denotes that bone measurements of an extant species were not available, whereas a dash indicates that the corresponding bone is unknown for a fossil taxon.

The Hammerschmiede clay pit has yielded more than 150 catalogued bird bones. Most of these belong to birds that lived in or near water, and in addition to the darter remains we identified at least five species of Anseriformes (waterfowl), a small species of Phalacrocoracidae (cormorants), and a fragmentary skull of a very large species of Gruidae (cranes). Remains of terrestrial or arboreal birds, by contrast, are very rare and include two species of Galliformes (landfowl), one or two species of Accipitridae (diurnal birds of prey), a passerine (Passeriformes) the size of the Eurasian Magpie (*Pica pica*; Corvidae), and a kingfisher (Alcedinidae) of about the size of the Collared Kingfisher (*Todiramphus chloris*), which is the first fossil record of an alcediniform bird from Europe. All of these fossils remain to be studied in detail, and in the present study we focus on the darter specimens.

## Material and methods

The studied specimens are deposited in the palaeontological collection of the University of Tübingen, Germany (GPIT), in the ornithological collection of Senckenberg Research Institute Frankfurt, Germany (SMF), and in the Bayerische Staatssammlung für Paläontologie und Geologie, Munich, Germany (BSPG). Of extant Anhingidae, skeletal material of *Anhinga anhinga*, *A*. *rufa*, and *A*. *melanogaster* (only trunk skeleton) was examined in the collection of Senckenberg Research Institute Frankfurt. Nomenclature of the extant species follows the IOC World Bird List at https://www.worldbirdnames.org.

All necessary permits were obtained for the described study, which complied with all relevant regulations (according to Bavarian law, no permits are required for palaeontological excavations; permission from the land owner has been obtained).

**Systematic palaeontology**

Aves Linnaeus, 1758

Suliformes Sharpe, 1891

Anhingidae Reichenbach, 1849

*Anhinga pannonica* (Lambrecht, 1916)

### Referred specimens

GPIT/AV/00138: cervical (5^th^ or 6^th^ praesacral) vertebra (HAM 5); GPIT/AV/00215: thoracic (20^th^ praesacral) vertebra (HAM 4); GPIT/AV/00223: partial right coracoid (HAM 4); GPIT/AV/00145: extremitas omalis of left coracoid (HAM 4); GPIT/AV/00217: right humerus (HAM 4); GPIT/AV/00127: distal and proximal ends of right ulna (HAM 5); GPIT/AV/00216: partial right carpometacarpus (HAM 4); GPIT/AV/00264: left femur lacking distal end (HAM 4); GPIT/AV/00220: proximal portion of right femur (HAM 4); GPIT/AV/00198: distal end of right tibiotarsus (HAM 4).

### Locality and horizons

Hammerschmiede clay pit near Pforzen, Allgäu region, Bavaria, Germany (47.923° N, 10.588° E); early late Miocene, Tortonian, MN 8, regional stratigraphic levels HAM 5 (11.62 Ma) and HAM 4 (11.44 Ma) [37].

### Measurements (in mm)

Cervical vertebra (GPIT/AV/00138), length: 31.7. Humerus (GPIT/AV/00217), length, 157.5; proximal width (from tuberculum ventrale to tuberculum dorsale), 21.0; distal width, 18.1. Carpometacarpus (GPIT/AV/00216), length as preserved, 72.7; estimated total length, ~77. Femur (GPIT/AV/00264), length as preserved, 62.3; estimated total length, ~65. Tibiotarsus (GPIT/AV/00198), distal width, 12.0.

### Taphonomic remarks

**Fig 1:**
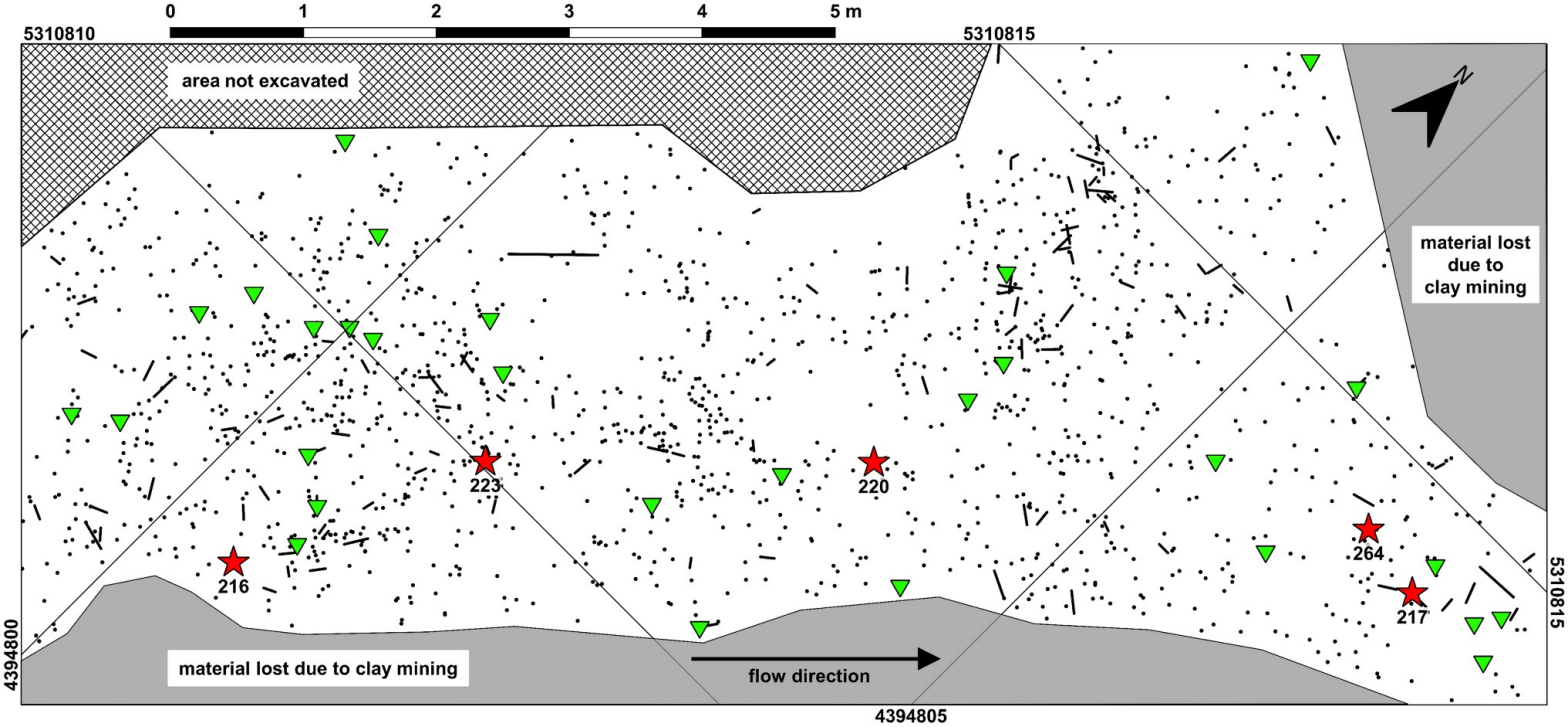
Section of the excavation plan Hammerschmiede level HAM 4 (excavation year 2019). Black dots represent vertebrate fossil specimens (black stripes denote the orientation of elongated objects). Excavated bird bones are shown with green triangles, and *Anhinga pannonica* bones, most probably belonging to the same individual, are highlighted with red stars (specimen GPIT/AV/00215 is a surface find from this area without coordinates). Associated bones of *A*. *pannonica* are arranged over a distance of nine meters parallel to the reconstructed flow direction (SSW-NNE) of the river.

In addition to numerous widely scattered finds of single bones and fragments, several partial mammal and turtle skeletons have been excavated in the Hammerschmiede locality. These include a male individual of the hominid *Danuvius guggenmosi* [38], as well as unpublished records of a boselaphine antelope (Bovidae: *Miotragocerus monacensis*), a chevrotain (Tragulidae: *Dorcatherium naui*), and a snapping turtle (Chelydridae: *Chelydropsis* sp.). Here, we also assume that six bones of *Anhinga pannonica* from HAM 4 belong to the same individual. These specimens–GPIT/AV/00215 (thoracic vertebra), GPIT/AV/00216 (partial right carpometacarpus), GPIT/AV/00217 (right humerus), GPIT/AV/00220 and GPIT/AV/00264 (right and left femur), as well as GPIT/AV/00223 (right coracoid)–were excavated over a distance of nine meters parallel to the reconstructed flow direction (SSW-NNE) of the HAM 4 river (Fig 1). The bones appear to have been sorted according to density and bone volume [43, 44], with the femora and the long humerus having been transported a longer distance than the smaller carpometacarpus and coracoid. No duplicate skeletal elements are present and all of the surrounding avian finds belong to other taxa, so that we hypothesize that dispersal of the darter remains goes back to one taphonomic event, which involved a single individual. Four further individuals of *A*. *pannonica* are represented by two bones each from HAM 4 (GPIT/AV/00145, GPIT/AV/00198 –found 15 m downstream and 45 meters upstream, respectively, of the above-mentioned associated remains) and HAM 5 (GPIT/AV/00127, GPIT/AV/00138).

### Description and comparisons

**Fig 2:**
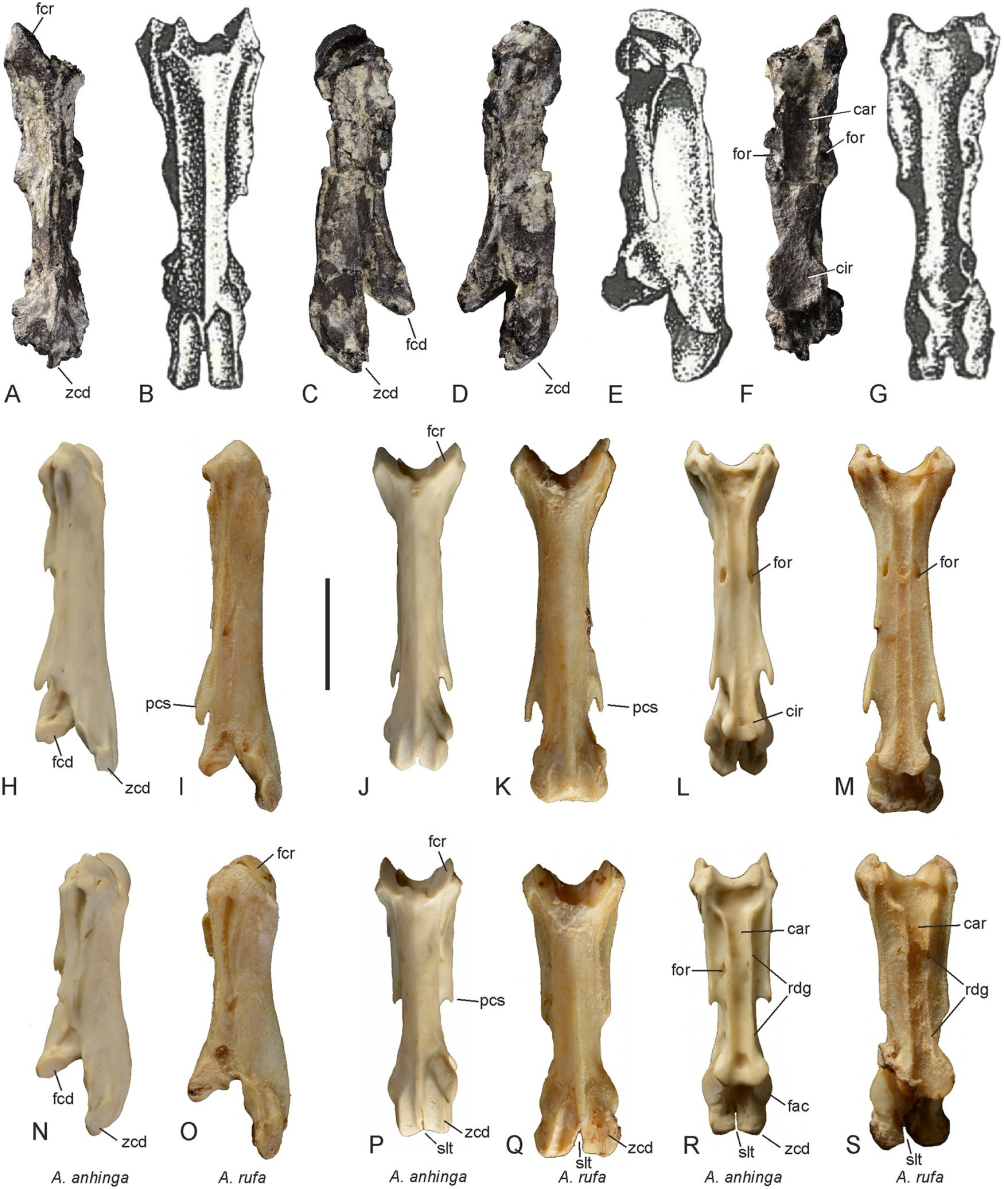
5^th^ or 6^th^ cervical vertebra of *Anhinga pannonica* from the late Miocene (MN 8) of the Hammerschmiede clay pit near Pforzen, Germany (**A**, **C**, **D**, **F**) in comparison to the holotype of *Anhinga pannonica* (**B**, **E**, **G**; from [25], original labeling removed) and the 4^th^ (**H**–**M**) and 6^th^ (**N**–**S**) cervical vertebrae of extant *A*. *anhinga* (SMF 9967) and *A*. *rufa* (SMF 9106). **A**, **B**, **J**, **K**, **P**, **Q**: dorsal view; **C**, right lateral view; **D**, **E**, **H**, **I**, **N**, **O**: left lateral view; **F**, **G**, **L**, **M**, **R**, **S**: ventral view. Abbreviations: car, sulcus caroticus; cir, circular expansion of vertebral corpus; fac, facies articularis; fcd, facies articularis caudalis; fcr, facies articularis cranialis; for, foramen delimited by the ridge-like shelf along the ventrolateral margin of the vertebral corpus; pcs, processus costalis; rdg, ridge bordering sulcus caroticus; slt, slit separating zygapophyses caudales; zcd, zygapophysis caudalis. The scale bar equals 10 mm (the size of the *A*. *pannonica* holotype is based on the measurements in [25]).

The cervical vertebra GPIT/AV/00138 exhibits a characteristic derived morphology that is only found in the cranial cervical vertebrae of the Anhingidae (Fig 2). Apart from being greatly elongated and narrow, GPIT/AV/00138 corresponds with the cranial cervical vertebrae of extant Anhingidae in that the processus costales are co-ossified with the corpus vertebrae and form ridge-like shelves along the ventrolateral margin of the corpus, which delimit a pair of lateral foramina (Fig 2F). The combination of these features is a diagnostic apomorphy of the Anhingidae. It is, however, less straightforward to identify the exact position of the fossil specimen within this series of cervical vertebrae, because it shows some differences to the cervical vertebrae of extant Anhingidae. The zygapophyses caudales project well beyond the facies articularis caudalis, and this derived morphology characterizes the 3^rd^ to 6^th^ (*A*. *anhinga*, *A*. *rufa*) or 3^rd^ to 7^th^ (*A*. *melanogaster*) cervical vertebrae of extant Anhingidae. The 3^rd^ vertebra of extant darters differs from the fossil in the presence of a shallow, ridge-like processus ventralis, which runs along the midline of the cranial portion of the vertebral corpus. In its proportions, GPIT/AV/00138 corresponds to the very elongate and narrow 4^th^ and 5^th^ vertebrae of extant darters, whereas the 6^th^ vertebra is proportionally shorter and stouter in extant darters (Fig 2). However, the fossil vertebra is only slightly longer (*A*. *anhinga*), as long as (*A*. *anhinga*, *A*. *rufa*) or shorter (*A*. *melanogaster*) than the 4^th^ and 5^th^ vertebrae of extant darters, which conflicts with the fact that the limb bones of the fossil are distinctly longer than those of extant Anhingidae (Table 1). Therefore, and because the zygapophyses caudales appear to have been separated by a narrow slit (they are fused along their midlines in the 4^th^ vertebra), we consider it most likely that GPIT/AV/00138 represents the 5^th^ or 6^th^ cervical vertebra. In size and morphology, GPIT/AV/00138 agrees well with the holotype of *A*. *pannonica* (identified as the 6^th^ cervical vertebra [25]) and a tentatively referred vertebra from the late Miocene of Tunisia (identified as the 7^th^ cervical vertebra [27]). With a length of 31.7 mm, GPIT/AV/00138 is slightly shorter than the holotype vertebra of *A*. *pannonica*, which has a length of 33 mm [25], whereas the vertebra from the late Miocene of Tunisia measures only 27.5 mm [27]. In lateral view, GPIT/AV/00138 is narrower than the holotype of *A*. *pannonica* and more closely resembles the vertebra from Tunisia [27]. However, as in the *A*. *pannonica* holotype and unlike in the Tunisian fossil, the zygapophyses caudales (the left one of which is broken in the fossil) are not fused along their midlines and appear to have been separated by a narrow slit. In ventral view, the corpus vertebrae has a keyhole-like shape and terminates in a circular expansion, which is situated caudal of a constriction of the vertebral corpus. The sulcus caroticus along the ventral surface of the corpus vertebrae is wider than in extant Anhingidae and is restricted to the cranial half of the vertebra, whereas it is laterally bordered by distinct ridges along the entire length of the vertebra in extant Anhingidae (Fig 2R). Cranially, the sulcus caroticus opens into a deep fossa. The facies articulares craniales correspond well with extant Anhingidae in their shape and orientation and are medially bordered by deep but narrow fossae.

**Fig 3:**
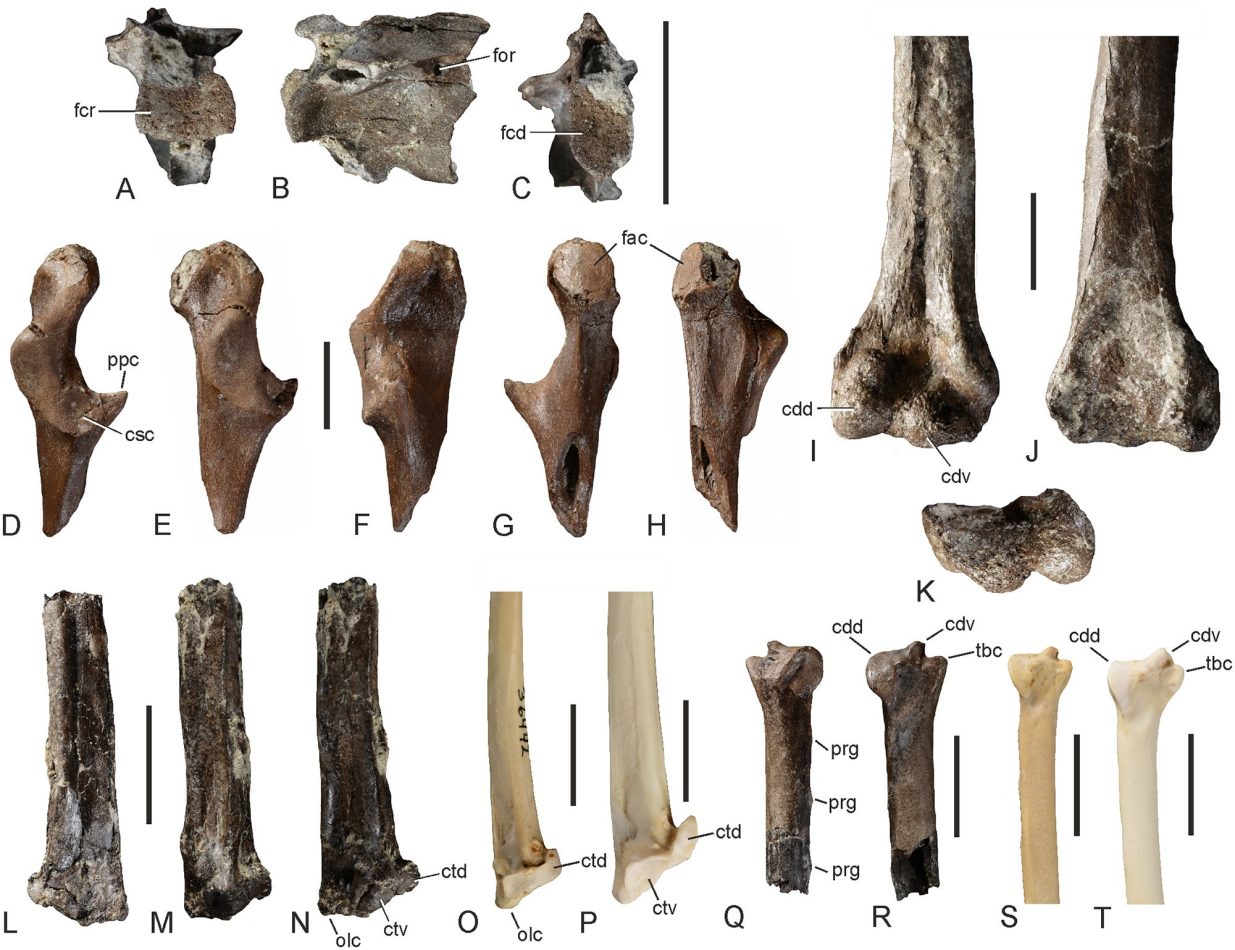
Various postcranial bones of *Anhinga pannonica* from the late Miocene (MN 8) of the Hammerschmiede clay pit near Pforzen, Germany. **A–C**, thoracic vertebra (GPIT/AV/00215) in cranial (**A**), left lateral (**B**), and caudal (**C**) view. **D–H**, extremitas omalis of left coracoid (GPIT/AV/00145) in dorsal (**D**), lateral (**E**), medial (**F**), ventromedial (**G**), and ventral (**H**) view. **I–K**, distal end of right humerus (GPIT/AV/00217) in cranial (**I**), caudal (**J**), and distal (**K**) view. **L–N**, proximal end of right ulna (GPIT/AV/00127) in caudodorsal (**L**), cranial (**M**), and cranioventral (**N**) view. **O**, *Anhinga anhinga* (SMF 9967), proximal end of right ulna in cranioventral view. **P**, *Phalacrocorax carbo* (Phalacrocoracidae; SMF 2939), proximal end of right ulna in cranioventral view. **Q**, **R**, distal end of right ulna (GPIT/AV/00127) in dorsal (**Q**) and ventral (**R**) view. **S**, *A*. *anhinga* (SMF 9967), distal end of right ulna in ventral view. **T**, *P*. *carbo* (SMF 2939), distal end of right ulna in ventral view. Abbreviations: cdd, condylus dorsalis; cdv, condylus ventralis; csc, cotyla scapularis; ctd, cotyla dorsalis; ctv, cotyla ventralis; fac, facies articularis clavicularis; fcd, facies articularis caudalis; fcr, facies articularis cranialis; for, foramen in vertebral corpus; olc, olecranon; ppc, processus procoracoideus; prg, papilla remigalis; tbc, tuberculum carpale. The scale bars equal 10 mm.

The thoracic vertebra GPIT/AV/00215 exhibits a saddle-shaped cranial articulation facet and a deeply concave caudal articulation facet with an oblong-oval shape (Fig 3A–3C). This unique combination of very differently-shaped cranial and caudal articulation surfaces identifies it as the 20^th^ vertebra of a darter (in the more cranial vertebrae of darters both articulation facets are saddle shaped, in the more caudal ones the cranial articulation facet is convex). As in extant Anhingidae, there is a small foramen on the lateral side of the corpus, just caudal of the processus transversus (Fig 3B). The processus ventrolaterales, which form distinct wings in extant Anhingidae, are broken and missing in the fossil specimen.

**Fig 4:**
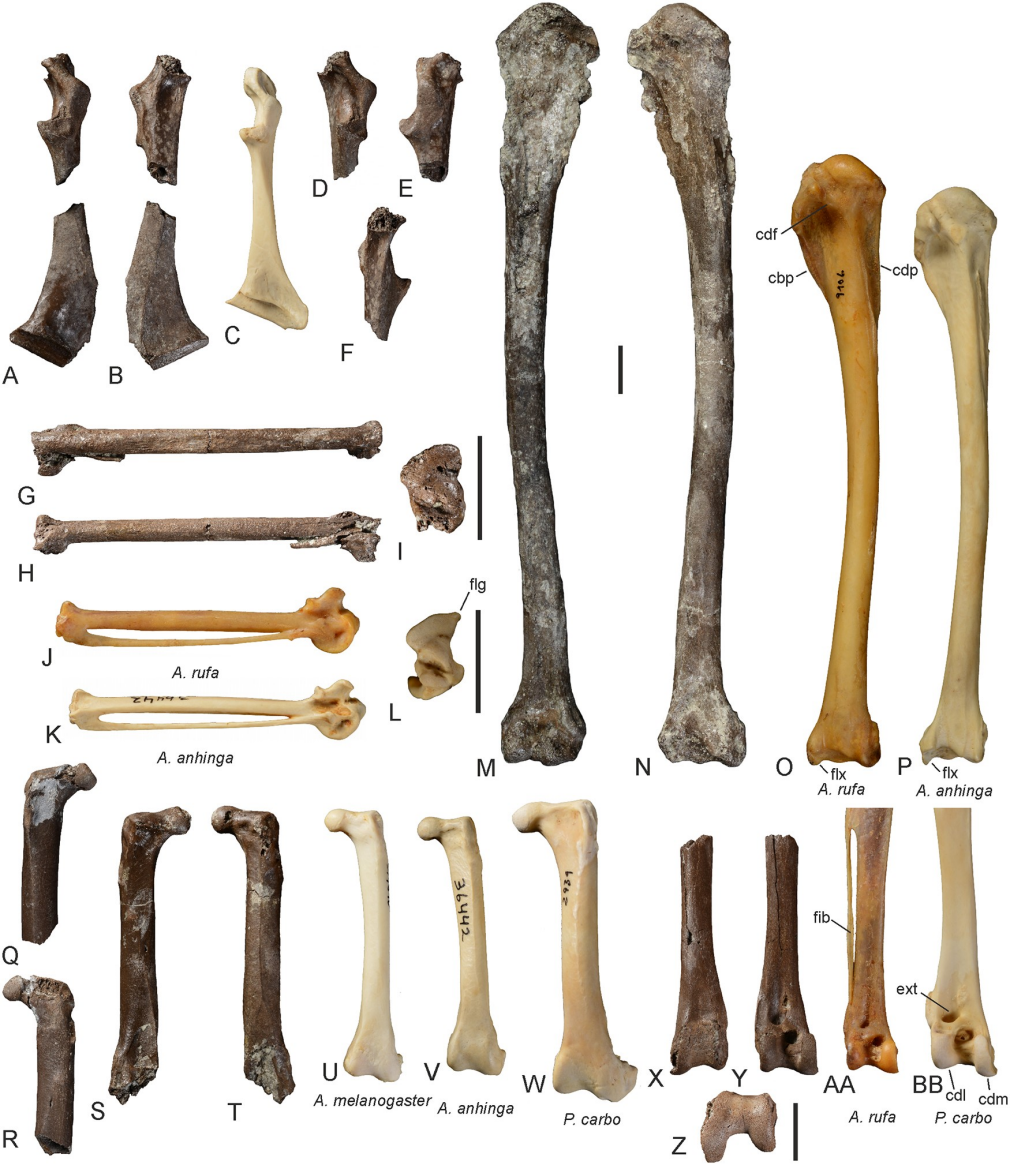
Major limb and pectoral girdle elements of *Anhinga pannonica* from the late Miocene (MN 8) of the Hammerschmiede clay pit near Pforzen, Germany. **A**, **B**, partial right coracoid (GPIT/AV/00223) in dorsal (**A**) and ventral (**B**) view. **C**, right coracoid of *Anhinga anhinga* (SMF 9967) in dorsal view. **D–F**, extremitas omalis of right coracoid GPIT/AV/00223 in dorsomedial (**D**), lateral (**E**) and ventromedial (**F**) view. **G–I**, partial right carpometacarpus (GPIT/AV/00216). in dorsal (**G**), ventral (**H**), and distal (**I**) view. **J**, right carpometacarpus of *A*. *rufa* (SMF 9106) in ventral view. **K**, **L**, right carpometacarpus of *A*. *anhinga* (SMF 9967) in ventral (**K**) and distal (**L**) view. **M**, **N**, right humerus (GPIT/AV/00217) in cranial (**M**) and caudal (**N**) view. **O**, right humerus of *A*. *rufa* (SMF 9106) in caudal view. **P**, right humerus of *A*. *anhinga* (SMF 9967) in caudal view. **Q**, **R**, proximal portion of right femur (GPIT/AV/00220) in cranial (**Q**) and caudal (**R**) view. **S**, **T**, left femur lacking distal end (GPIT/AV/00264) in caudal (**S**) and cranial (**T**) view. **U**, left femur (cranial view) of *A*. *melanogaster* (SMF 19890). **V**, left femur (cranial view) of *A*. *anhinga* (SMF 9967). **W**, left femur (cranial view) of *Phalacrocorax carbo* (Phalacrocoracidae; SMF 2939). **X–Z**, distal end of right tibiotarsus (GPIT/AV/00198) in caudal (**X**), cranial (**Y**), and distal (**Z**) view. **AA**, distal end of right tibiotarsus of *A*. *rufa* (SMF 9106) in cranial view. **BB**, distal end of right tibiotarsus of *P*. *carbo* (SMF 2939) in cranial view. Abbreviations: cbp, crita bicipitalis; cdf, crus dorsale fossae; cdl, condylus lateralis; cdm, condylus medialis; cdp, crista deltopectoralis; ext, sulcus extensorius; fib, fibula; flg, ridge-like flange on distal end of os metacarpale majus; flx, processus flexorius. The scale bars equal 10 mm; same scale bar for all images except **I**, **L**, and **Z**.

The coracoid (Figs 3D–3H, 4A–4F) differs from that of extant Anhingidae (Fig 4C) in that the processus acrocoracoideus is dorsoventrally narrower and the facies articularis clavicularis longer in sterno-omal direction. The facies articularis scapularis is slightly concave, whereas it is essentially flat in extant Anhingidae. As in *Meganhinga chilensis* [5], the processus procoracoideus is proportionally longer than in crown group Anhingidae and its tip is more pointed than the more “knob-like” processus procoracoideus of extant darters. The medial margin of the extremitas omalis forms a sharp ridge. On the ventral surface of the extremitas omalis, just omal of the facies articularis clavicularis, there is a distinct fossa, which is also present in extant Anhingidae, but which is absent in the Phalacrocoracidae. The extremitas sternalis resembles that of extant Anhingidae.

Several partial humeri were—tentatively, at least—referred to *A*. *pannonica* [23, 27, 30, 33, 34], but GPIT/AV/00217 (Fig 4M and 4N) is the first nearly complete humerus assigned to the species (on the proximal end of the bone, the tuberculum ventrale is broken and the margins of the crista deltopectoralis and crista bicipitalis exhibit some damage). In size, the humerus from the Hammerschmiede clay pit corresponds well with the proximal humeri assigned to *A*. *pannonica* by previous authors [27, 30, 34]. Mlíkovský [33] did not publish measurements for the partial humeri from Götzendorf in Austria and the plate lacks a scale. However, the more complete specimen has a length of 125 mm (U. Göhlich, pers. comm.) and was thus depicted in original size. Because the fossil lacks about one fifth of its proximal end, the original length of the bone was about 155 mm and compares well with the length of the humerus from the Hammerschmiede clay pit. The new fossil has similar overall proportions to the humerus of extant Anhingidae and is distinguished from the humerus of the Phalacrocoracidae in, e.g., the proportionally longer crista deltopectoralis (which reaches much farther distally than the crista bicipitalis), the poorly developed crus dorsale fossae, which does not overhang the fossa pneumotricipitalis, as well as the more expanded distal end. Furthermore as in extant Anhingidae, the crista bicipitalis is sheet-like with a flat cranial surface (convex in the Phalacrocoracidae) and the sulcus transversus is shallower than in the Phalacrocoracidae. The long shaft of the bone is slightly sigmoidally curved. On the distal end, the processus flexorius does not form a marked, distally projecting rim, which is present in *Anhinga grandis* and also found in extant Anhingidae (Fig 4O and 4P). However, the corresponding portion of the bone exhibits some damage and we hypothesize that this rim is broken in GPIT/AV/00217. Otherwise, the distal end of the specimen closely resembles the distal humerus of extant Anhingidae, but owing to the fact that the bone surface is eroded, many osteological details, such as the shape of the fossa musculi brachialis, cannot be discerned.

Of the ulna (GPIT/AV/00127), the proximal and distal ends are preserved (Fig 3L–3N, 3Q and 3R). Whereas the distal end is undistorted, the proximal end is dorsoventrally compressed, so that its original shape is deformed. However, it can still be observed that the tuberculum ligamenti collateralis ventralis is similar to that of extant darters in size and position, whereas it is shallower and more distally situated in the Phalacrocoracidae. The cotyla dorsalis is likewise similar to that of extant darters in its proportions; unlike in the Phalacrocoracidae it does not have a hook-like shape. The preserved distal portion of the shaft allows recognition of three papillae remigales. As in extant darters, the distal tip of the condylus ventralis, on the distal end of the bone, is strongly projected and is separated from the tuberculum carpale by a marked notch; unlike in the Phalacrocoracidae it is not cranially expanded and confluent with the tuberculum carpale. The condylus dorsalis has a somewhat more convex profile than in extant Anhingidae, whereas the cranial margin of the tuberculum carpale is straighter than in extant Anhingidae. In size, the distal end of the bone resembles a distal ulna that was tentatively assigned to *Anhinga grandis* [17], but the tuberculum carpale is narrower and more pointed than in the latter fossil and extant Anhingidae. On the dorsal surface, just proximal of the condylus ventralis, the bone has a rugose surface and exhibits several small pneumatic foramina.

The carpometacarpus GPIT/AV/00216 lacks the os metacarpale minus, the processus extensorius and the proximal portion of the trochlea carpalis (Fig 4G–4I). As far as comparisons are possible, the remaining sections of the bone closely correspond with a carpometacarpus referred to *A*. *pannonica* [25]. With an estimated length of ca. 77 mm, the carpometacarpus from the Hammerschmiede clay pit is, however, slightly longer than the latter specimen, for which a length of 73 mm was given [25]. In size and morphology, GPIT/AV/00216 resembles the carpometacarpus of *Phalacrocorax carbo* (Phalacrocoracidae), whereas the corresponding bone of extant Anhingidae has a more ridge-like flange on the distal end of the os metacarpale majus (Fig 4I and 4L). The bone is, however, too large to belong to the undescribed cormorant in the avian material from the Hammerschmiede clay pit and is here referred to *A*. *pannonica* based on its resemblance to the holotype of this species.

The femur (Fig 4Q–4T) closely corresponds with that of extant Anhingidae and is much more elongated than the stout femur of the Phalacrocoracidae. As in extant Anhingidae and unlike in the Phalacrocoracidae, the caput femoris is somewhat proximally directed and projects beyond the facies articularis antitrochanterica. The muscle insertion scars on the lateral surface of the proximal end, for musculus obturatorius lateralis and medialis, m. caudofemoralis, and m. ischiofemoralis ([45]: Fig 4O), closely match those of extant Anhingidae. On the lateral surface of the broken distal end of the more complete femur GPIT/AV/00264, a raised bulge presumably for musculus flexor perforans digiti II ([45]: Fig 4O) and a pit for the insertion of musculus flexor perforans et perforatus digiti II are preserved. The femur of *A*. *pannonica* is more elongated than the stout femora of the larger *Macranhinga paranensis* [4] and *M*. *ranzii* [6].

The tibiotarsus (Fig 4X–4Z) is likewise very similar to that of extant Anhingidae. As in other darters, the sulcus extensorius is centrally situated, whereas it is positioned more laterally in the Phalacrocoracidae. As in other Anhingidae but unlike in the Phalacrocoracidae, the distal end of the fibula seems not to have been fused to the tibiotarsus and the condylus medialis is less strongly protruding in distal direction (Fig 4BB).

## Discussion

### The fossil record of *Anhinga pannonica*

The specimens from the Hammerschmiede clay pit are assigned to *Anhinga pannonica*, which is the only previously described species of the Anhingidae from Europe. The cervical vertebra described in the present study agrees well with the holotype of *A*. *pannonica* in size and morphology (Fig 2), and the age of the fossils from the Hammerschmiede clay pit (11.62–11.44 Ma) is close to that of the holotype of *A*. *pannonica*, from Brusturi/Tataros (~10 Ma; Pannonian E, [46]).

Some bones of the Anhingidae, such as the cervical vertebrae, exhibit a unique derived morphology that makes an unambiguous identification straightforward. However, most limb and pectoral girdle elements of darters closely resemble those of cormorants (Phalacrocoracidae). Even though consistent differences exist, which allow a clear distinction of most major bones of darters and cormorants [15, 18], some Neogene darter fossils were initially assigned to the Phalacrocoracidae. This is true for *Anhinga* (“*Phalacrocorax*”) *subvolans* from the early Miocene of North America [18], and here we show that putative cormorants from the Miocene of Europe likewise represent a misidentified record of *A*. *pannonica*.

**Fig 5:**
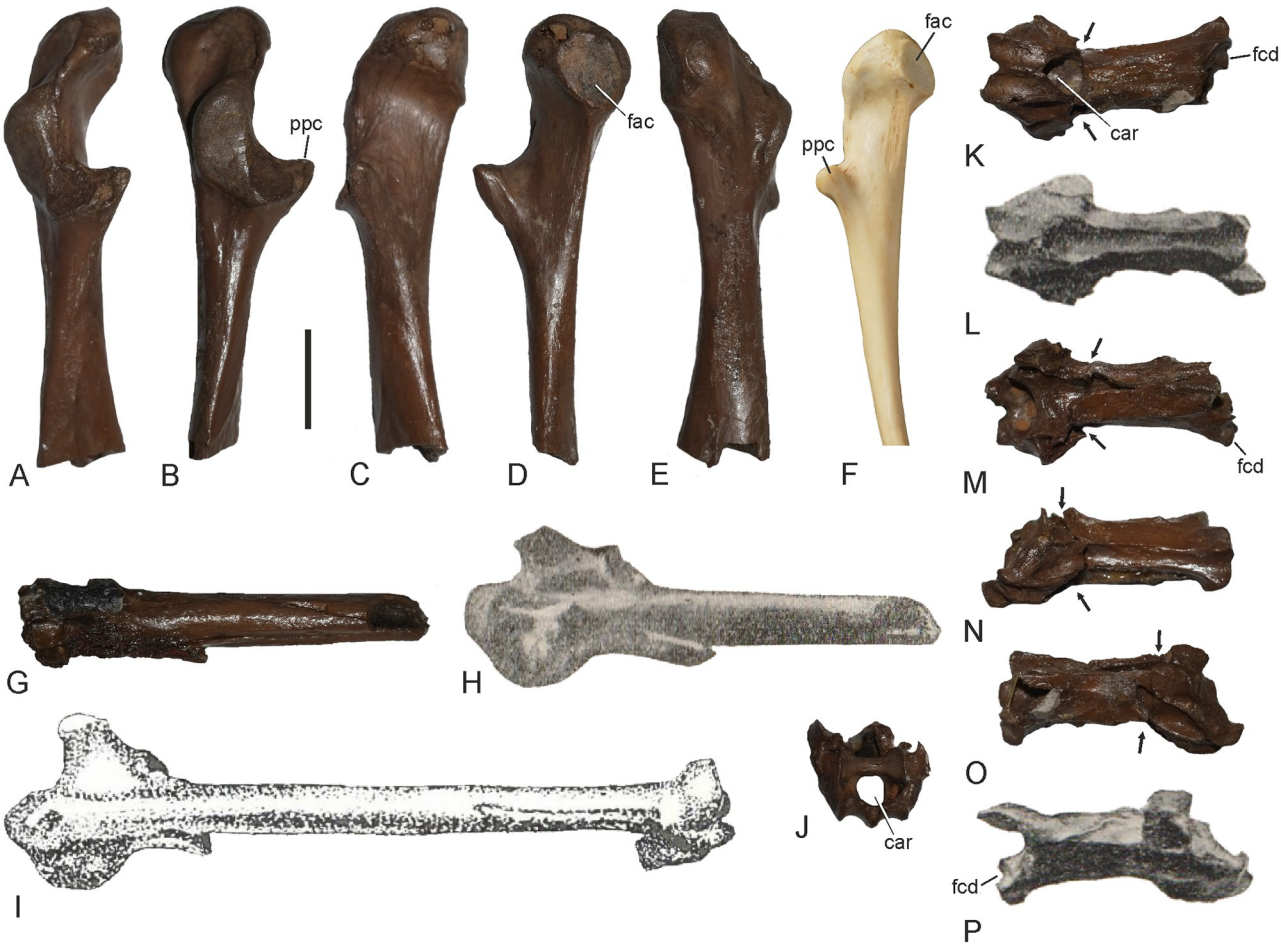
Fossils of *Anhinga pannonica* from the middle Miocene (MN 5) of Regensburg-Dechbetten near Regensburg, Germany. **A**–**E**, Left coracoid (BSPG 2008 LI 671; holotype of *Phalacrocorax praecarbo* von Ammon, 1918) in dorsal (**A**), dorsolateral (**B**), medial (**C**), ventromedial (**D**), and ventral (**E**) view. **F**, Left coracoid of the extant *Anhinga anhinga* (SMF 9967) in medial view. **G**, Partial left carpometacarpus (BSPG 2008 LI 607; holotype of *Ardea brunhuberi* von Ammon, 1918) in cranioventral view; **H**, the former specimen as it was figured by von Ammon [47]. **I**, referred left carpometacarpus of *A*. *pannonica* from the late Miocene of Brusturi in Romania (mirrored to ease comparisons and original labeling removed; from [25]). **J**–**P**, cervical vertebra (BSPG 2008 LI 676; holotype of *Botaurites avitus* von Ammon, 1918) in different views (**J**: cranial; **K**: ventral [cranial portion]/right lateral [caudal portion]; **M**: dorsal [cranial portion]/left lateral [caudal portion]; **N**: left lateral [cranial portion]/ventral [caudal portion]; **O**: right lateral [cranial portion]/dorsal [caudal portion]); **L** and **P** are from [47] and show the original condition of the bone in ventral (**L**) and right lateral (**P**) view; the arrows in **K** and **M**–**O** indicate the line of breakage separating the incorrectly reassembled cranial and distal portions of the vertebra. Abbreviations: car, canalis caroticus; fac, facies articularis clavicularis; fcd, facies articularis caudalis; ppc, processus procoracoideus. The scale bar equals 10 mm (the size of **H**, **I**, **L**, and **P** is based on measurements in [25] and [47].

The specimens in question (Fig 5) stem from the early middle Miocene (MN 5) locality of Regensburg-Dechbetten (Germany) and were described by von Ammon [47] as a new cormorant species, *Phalacrocorax praecarbo* von Ammon, 1918 (omal extremity of a coracoid), and two new species of herons: *Ardea brunhuberi* von Ammon, 1918 (proximal carpometacarpus) and *Botaurites avitus* von Ammon, 1918 (cervical vertebra). The type material of these species is in the collection of Bayerische Staatssammlung für Paläontologie und Geologie in Munich and not in Senckenberg Research Institute Frankfurt, as erroneously indicated by Mlíkovský ([48]: 71), who also mistakenly considered the locality to be from the Mammalian Neogene Zones MN 7–8.

Brodkorb [49] hypothesized that the carpometacarpus described as “*Ardea brunhuberi*” is from a cormorant and synonymized *Phalacrocorax praecarbo* with *Phalacrocorax* (“*Ardea*”) *brunhuberi*, which is unfortunate, because the holotype of *A*. *brunhuberi* was badly damaged after von Ammon’s [47] description (Fig 5G and 5H) [50]. Olson ([51]: 167) subsequently noted that the vertebra, which constitutes the holotype of *Botaurites avitus* von Ammon, 1918 and was not considered by Brodkorb [49], is “almost certainly from a cormorant of the same size, so that this name likewise is best synonymized with *Phalacrocorax brunhuberi*.” This vertebra was also broken after von Ammon’s [47] publication, and the cranial and caudal portions of the bone are incorrectly glued and twisted at 90 degrees (Fig 5J–5P). However, it can still be discerned that the processus carotici are ankylozed along their midline and form a canalis caroticus, which is a derived characteristic of the Ardeidae (hence, von Ammonʼs [47] identification), Anhingidae, and a few other taxa of the waterbird clade (Aequornithes), but which is absent in the Phalacrocoracidae. In other features, the holotypical vertebra of *B*. *avitus* also closely resembles the 9th or 10^th^ praesacral vertebra of a darter. The coracoid that constitutes the holotype of *P*. *praecarbo* (Fig 5A–5E) is very similar to the coracoids of *A*. *pannonica* from the Hammerschmiede clay pit and differs from the coracoid of the Phalacrocoracidae in the shorter and more rounded processus acrocoracoideus and the slightly concave cotyla scapularis. Accordingly, we transfer the vertebra, coracoid and carpometacarpus described by von Ammon [47] to the Anhingidae and synonymize *Ardea brunhuberi* von Ammon, 1918, *Phalacrocorax praecarbo* von Ammon, 1918, and *Botaurites avitus* von Ammon, 1918 with *Anhinga pannonica* Lambrecht, 1916.

**Fig 6:**
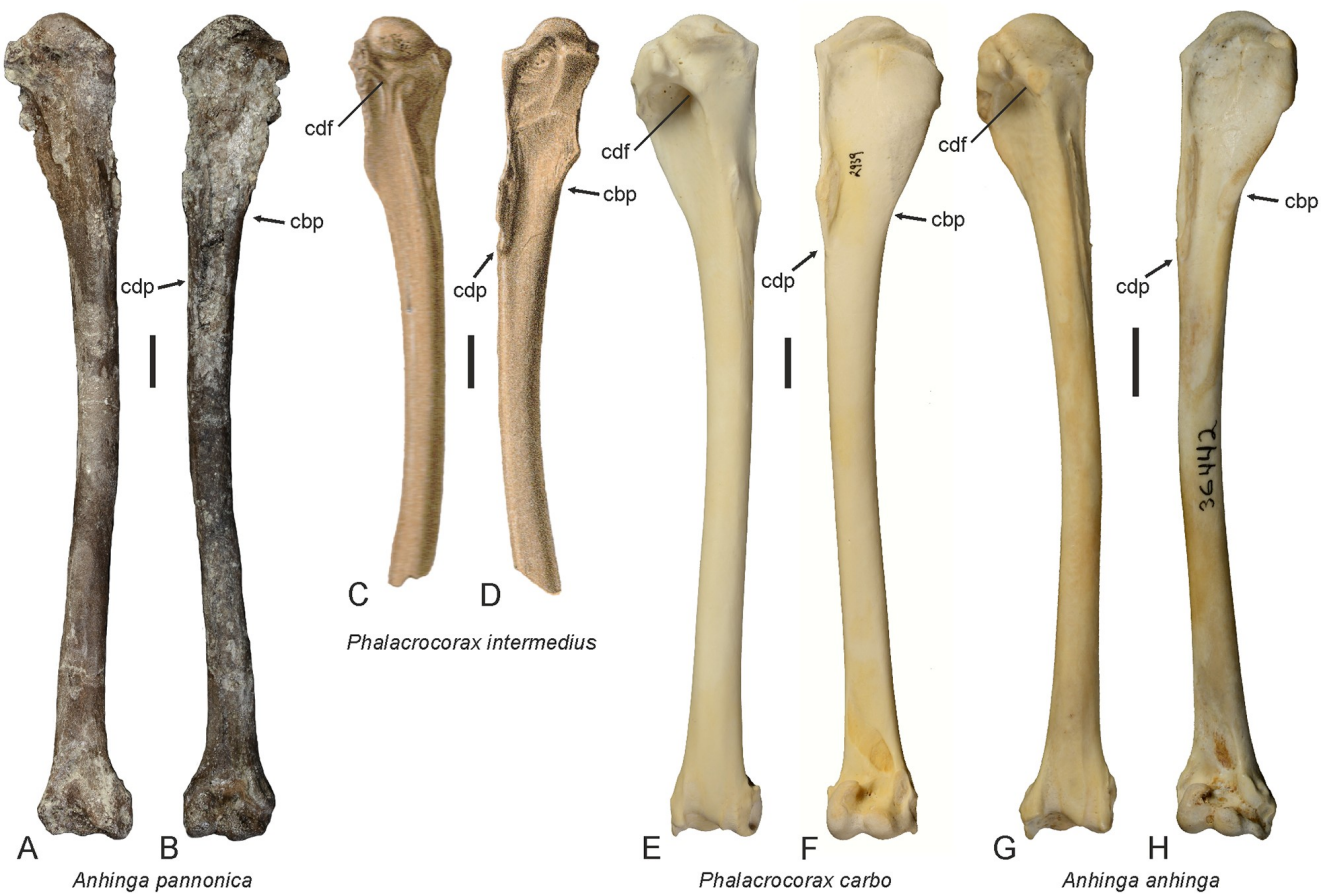
Humeri of extant and fossil Anhingidae and Phalacrocoracidae. **A**, **B**, *Anhinga pannonica* from the Hammerschmiede clay pit (GPIT/AV/00217), **C**, **D**, *Phalacrocorax intermedius* from the early Miocene of France; from ([52]: pl. 43), **E**, **F**, extant *Phalacrocorax carbo* (SMF 2939), and **G**, **H**, extant *Anhinga anhinga* (SMF 9967); the bones are shown in caudal (**A**, **C**, **E**, **G**) and cranial (**B**, **D**, **F**, **H**) view. The arrows indicate the distal ends of the crista bicipitalis and crista deltopectoralis. Note the long crista deltopectoralis and poorly developed crus dorsale fossae of *Phalacrocorax intermedius*. Abbreviations: cbp, crista bicipitalis; cdf, crus dorsale fossae; cdp, crista deltopectoralis. The scale bars equal 10 mm; the size of *P*. *intermedius* was inferred from ([52]: pl. 43), which depicts the fossil in natural size).

Mlíkovský [48] synonymized *Phalacrocorax brunhuberi* (von Ammon, 1918) with *Phalacrocorax intermedius* Milne-Edwards, 1867, which is based on an incomplete proximal humerus from the early Miocene (MN 4) of France. This fossil, which was figured by Milne-Edwards ([52]: pl. 43, Figs 8–11), is of similar size to the humerus of *A*. *pannonica* and differs from the humerus of extant Phalacrocoracidae in the proportionally longer crista deltopectoralis, which reaches distally well beyond the distal end of the crista bicipitalis and in the less developed crus dorsale fossae (Fig 6). We consider it likely that *P*. *intermedius* is another misidentified darter, in which case *P*. *intermedius* Milne-Edwards, 1867 may be a senior synonym of *Anhinga pannonica* Lambrecht, 1916. However, a definitive taxonomic assessment of the species is only possible once the holotype has been directly examined. If anhingid affinities of *P*. *intermedius* can be shown, these need also to be considered for a pelvis with associated thoracic vertebrae from the early Miocene (MN 3) of the Czech Republic and a carpometacarpus from the middle Miocene (MN 5) of Austria, which were assigned to this species [53, 54].

As detailed above, the size of the humerus from the Hammerschmiede clay pit corresponds well with humeri of *A*. *pannonica* from Hambach in Germany [34] and Götzendorf in Austria [33]. With an estimated length of ca. 155 mm, the humerus of an unidentified darter from the latest Miocene of Libya [31] likewise has almost the same length as the fossil from the Hammerschmiede clay pit. A humerus from the late Miocene of Chad, which lacks only a part of the proximal shaft section, was tentatively assigned to *A*. *pannonica* and its total length was estimated at 167 mm [23]; although this bone is larger than other humeri assigned to *A*. *pannonica*, the size difference is not greater than that observed in extant Anhingidae (Table 1).

**Fig 7:**
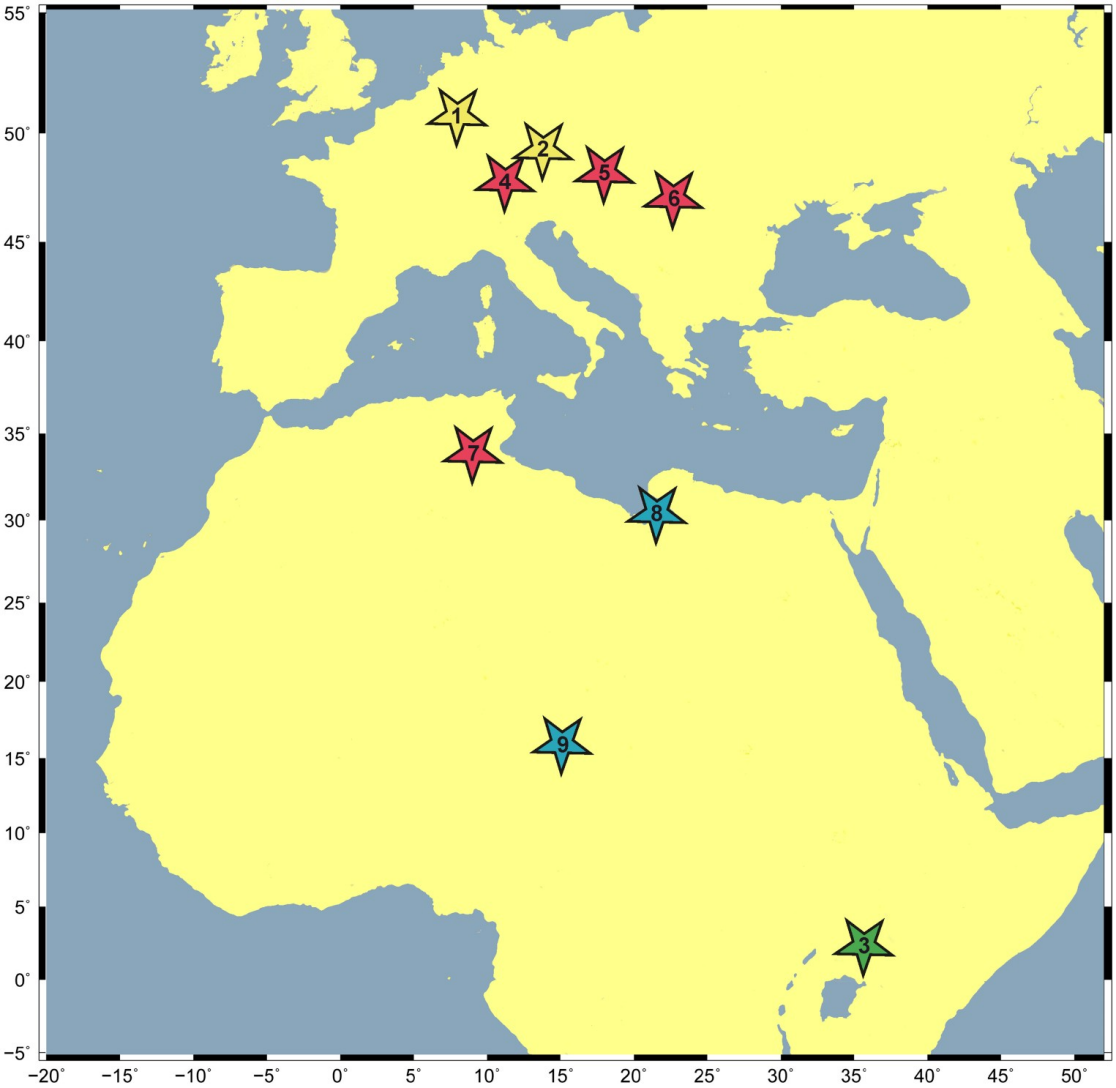
Geographic and stratigraphic distribution of *Anhinga pannonica* in Europe and Africa. Early middle Miocene (yellow stars): 1 –Hambach (Germany), 2 –Regensburg-Dechbetten (Germany). Late middle Miocene (green star): 3 –Ngorora Formation (Kenya). Early late Miocene (red stars): 4 –Hammerschmiede (Germany), 5 –Götzendorf (Austria), 6 –Brusturi/Tataros (Romania), 7 –Beglia Formation (Tunisia). Late late Miocene (blue stars): 8 –Sahabi Formation (Libya), 9 –Toros-Menalla (Chad).

The Asian fossils of *Anhinga* cf. *pannonica* from Pakistan [28] and Thailand [29], by contrast, fall within the size range of extant darters, which suggests that they do not belong to *A*. *pannonica*. The distal width of the tibiotarsus of the species from Thailand measures 10.7 mm [29], which is less than in *A*. *pannonica* (12.0 mm; this study), and it was assumed that “the size of the elements […] is somewhat intermediate between the size of the recent *A*. *anhinga* and the size of *Anhinga* of the *melanogaster* group” ([29]: 121). The putative *A*. *pannonica* bones from the late Miocene of Pakistan were considered to be “slightly larger than those of *A*. *anhinga*” ([28]: 56) and the specimens are of similar size to darter remains from the late Pliocene of India [24]. We therefore conclude that only the records of *A*. *pannonica* from Europe (Romania, Austria, and Germany) and Africa (Kenya, Tunisia, Libya, and Chad) can be referred to the species (Fig 7) and that the taxonomic identity of the Asian material needs to be revised.

With nine occurrences from middle and late Miocene sediments of Europa and Africa (Fig 7), *Anhinga pannonica* exhibits a long stratigraphic occurrence over 10 million years as well as a large geographic distribution, stretching from the equator (Ngorora Formation) to 50° northern latitudes (Lower Rhine Basin). Its oldest records at the beginning of the middle Miocene from Regensburg-Dechbetten (~16 Ma) and Hambach 6 (~15 Ma) represent the northernmost localities. During the early late Miocene (11.6–9.8 Ma, Vallesian and early Tortonian), a period of considerably increased hydrologic cycle and spread of freshwater habitats in Europe and North Africa known as the first washhouse climate period [55], this species shows a wide circum-Mediterranean distribution (Fig 7). Its latest occurrences are documented from the early Messinian (6–7 Ma) of North Africa. The significant Northern Hemispheric meridional distribution is in accordance with the strongly reduced hemispheric temperature gradient during most of the Miocene, where topical temperatures prevail in mid-latitudinal southern Europe from the Langhian to the end of Tortonian (16–7.5 Ma) [56].

### The size of *Anhinga pannonica* and the evolution of large-sized darters

Lambrecht [25] noted that *A*. *pannonica* was larger than *A*. *anhinga*, but owing to the limited material available to him, he did not further specify the size difference. Other authors considered the species to be “the size of a large *Anhinga anhinga*” ([27]: 48), “somewhat larger” than *A*. *rufa* ([34]: 115), or to fall “into the upper part of the range of extant *A*. *melanogaster*” ([31]: 114). The new fossils show that the size of *A*. *pannonica* has been underestimated by earlier authors and, with a length of 157.5 mm, the humerus is significantly longer than that of all extant Anhingidae and of similar length to the humerus of *A*. *grandis* from the late Miocene of North America (Table 1). Compared with extant darters, the humerus and all other sufficiently complete limb bones of *A*. *pannonica* are about 15% larger than those of extant Anhingidae and approach the size of the corresponding bones of the Great Cormorant, *Phalacrocorax carbo* (Figs 4 and 6).

The least circumference of the femur shaft (C_F_) allows an assessment of the body mass (M) of a bird, with log_10_ M being proportional to log_10_ C_F_ [57]. For foot-propelled diving birds, mean log_10_ M = 2.938 and mean log_10_ C_F_ = 1.209 [57, 58]. With a least femur shaft circumference of 22.4 mm (GPIT/AV/00264), log_10_ C_F_ is 1.350 for *Anhinga pannonica*, which results in a mass estimate of about 3.3 kg. This value distinctly exceeds the body mass of extant darters, which is 1–1.8 kg [1], but it is less than the weight estimates of 5.4 to 25 kg for some of the extinct giant South American darters [8, 58].

Mlíkovský ([33]: 98) commented on the large size of *A*. *pannonica* and compared the species with *A*. *grandis*, noting that it “is worth mentioning that while *Anhinga pannonica* belonged to the Old World anhingas, *Anhinga grandis* was a representative of the New World anhingas, so that both these phyletic lines of anhingas parallelly developed large-sized forms during the late Miocene”. Actually, however, the phylogenetic affinities of both *A*. *pannonica* and *A*. *grandis* are poorly constrained, and we note that *A*. *pannonica* can hardly be differentiated from the similar-sized (Table 1) *A*. *grandis* based on the published descriptions and photographs of the latter species. There even remains a possibility that *A*. *grandis* is a junior synonym of *A*. *pannonica*, but definitive taxonomic conclusions have to await a direct examination of the *A*. *grandis* material.

The sole published phylogeny of fossil Anhingidae [59] includes a single crown group representative, the New World *A*. *anhinga*, and is mainly based on features of the tarsometatarsus and pelvis. The tarsometatarsi of extant darters differ in the morphology of the hypotarsus, which exhibits a closed canal for the tendon of musculus flexor perforans et perforatus digiti 2 in the New World anhinga, *A*. *anhinga*, whereas this tendon is guided by an open sulcus in the three Old World species [60, 61]. It was hypothesized that the presence of a canal in *A*. *grandis* suggests close affinities between this fossil species and *A*. *anhinga* [16]. The canal for musculus flexor perforans et perforatus digiti 2 is absent in the oldest known darter, *Anhinga walterbolesi* from the late Oligocene/early Miocene of Australia [19], which may indicate that its absence is indeed a plesiomorphic trait of crown group Anhingidae. Unfortunately, the tarsometatarsus is unknown for *A*. *pannonica* (as noted above, partial tarsometatarsi from the Miocene of Thailand and Pakistan probably do not belong to the species), and we can neither exclude the possibility that *A*. *pannonica* and *A*. *grandis* are closely related nor the alternative hypothesis that a large size evolved convergently in New World and Old World darters. Irrespective of their exact interrelationships, however, large-sized Anhingidae appear to have been widespread in the Miocene and Pliocene of Europe, Africa, and the Americas. At least in Africa and South America, they coexisted with smaller forms, whose size was within the range of extant darters [22, 23, 62] or even fell below that of the smallest extant species (*Anhinga minuta* [6]).

The earliest definitive records of *A*. *pannonica* are the specimens from Regensburg-Dechbetten and Hambach in Germany (MN 5; ca. 15.2–16 Ma), but depending on the affinities of “*Phalacrocorax*” *intermedius* (see above), the species possibly already occurred in the early Miocene (MN 4 or even MN 3) of France and the Czech Republic, about 16–20 Ma. In Europe, *A*. *pannonica* therefore existed for at least 5 million years before it disappeared towards the early late Miocene (MN 10; 9–10 Ma). There is no Paleogene record of darters in Europe and the occurrence of *A*. *pannonica* in Europe is likely to be the result of an early or middle Miocene dispersal. Mayr ([14]: 183) hypothesized that it “may go back to a Miocene dispersal from Africa, which probably also led to range extensions of other African bird groups”. Removal of the Asian fossils from the record of *A*. *pannonica* seems to support this hypothesis, but the existence of similar-sized darters in North America places a caveat on premature biogeographic hypotheses.

With an age of about 8.5 Ma (early Hemphillian [16]), the North American fossils of *A*. *grandis* are roughly coeval to or only slightly younger than the latest European records of *A*. *pannonica*, but a tentatively referred ulna from the middle Miocene of Colombia was dated at 14.6–16.1 Ma [17] and therefore corresponds in age to the *A*. *pannonica* fossils from MN 5. A future revision of the early Miocene material assigned to *Phalacrocorax intermedius* and phylogenetic analyses including *A*. *grandis* and *A*. *pannonica* may eventually confirm an Old World origin of very large Anhingidae and their dispersal into the New World, but at present no well-founded biogeographic scenarios can be established.

Calibrated molecular data suggest that Old World and New World Anhingidae diverged 19–22 Ma [2]. This divergence estimate postdates the occurrence of the oldest known darter, *A*. *walterbolesi* from Australia, which stems from strata that are 24–26 million years old and which is distinguished from crown group Anhingidae in a plesiomorphic hypotarsus morphology [19]. *A*. *walterbolesi* was slightly larger than the largest extant Anhingidae, which possibly suggests that darters underwent a size decrease in their evolution. The smallest extant darter is the New World *A*. *anhinga*, but an even smaller species, *A*. *minuta*, occurred in the late Miocene/early Pliocene of Brazil [6]. The known records of *A*. *pannonica* and other very large darters are younger than the presumed divergence of Old World and New World darters. Even though a size decrease may have occurred in New World species after darters dispersed into the Americas, any considerations on trends in the evolution of the Anhingidae have to remain speculative in the absence of a robust phylogenetic framework.

To reduce buoyancy when diving, darters have a highly wettable plumage, which makes them prone to temperature loss during and after dives. Accordingly, these birds spend much time sunning on exposed perches, where they adopt a characteristic posture with widely spread wings [1]. These physiological constraints confine the extant distribution of darters to subtropical and tropical zones, and because a larger size results in a more favorable (with regard to heat loss) surface to volume ratio in endothermic animals, it may have been positively selected for in Neogene darters. This is in agreement with the reconstructed palaeoclimate of the Hammerschmiede locality, which was warm-subtropical with mean annual temperatures probably over 20°C. The extinction of darters in Europe is likely to have been due to climatic cooling in the late Neogene, but the reasons for the disappearance of large-sized Anhingidae in the Pliocene of Africa and in the Plio-Pleistocene of South America remain elusive.
